# High-Dose Intravenous Vitamin C and Mistletoe Therapy in Breast Cancer: A Prospective Cohort Study on Overall Survival in Palliative Care

**DOI:** 10.3390/cancers18193094

**Published:** 2026-09-23

**Authors:** Donghun Lee, Ha-Eun Ryu, Byoungjin Park, Chang Hwan Yeom, Seok-Jae Heo, Dong-Hyuk Jung

**Affiliations:** 1 Department of Family Medicine, Severance Hospital, Seoul 03722, Republic of Korea; fkh28@yuhs.ac; 2 Department of Family Medicine, Yonsei University College of Medicine, Seoul 03722, Republic of Korea; rahahaha@yuhs.ac (H.-E.R.); bjpark96@yuhs.ac (B.P.); 3 Department of Family Medicine, Yongin Severance Hospital, Yongin 16995, Republic of Korea; 4 Department of Family Medicine, Yeom Chang Hwan Hospital, Seoul 05700, Republic of Korea; lymphych@yuhs.ac; 5 Division of Biostatistics, Department of Biomedical Systems Informatics, Yonsei University College of Medicine, Seoul 03722, Republic of Korea

**Keywords:** breast cancer, palliative care, high-dose vitamin C, mistletoe, complementary therapy, survival analysis

## Abstract

**Background:** High-dose intravenous vitamin C (IVC) and mistletoe therapy are widely used in palliative care as complementary interventions to relieve symptoms and improve quality of life. However, prospective evidence regarding their impact on overall survival in breast cancer remains limited. We investigated the longitudinal association between individual complementary therapies and overall mortality in female breast cancer patients. **Methods:** This prospective cohort study analyzed 748 female breast cancer patients. To account for time-varying exposure and prevent immortal time bias, Cox proportional-hazards regression models incorporating time-dependent covariates with a counting-process structure were used to estimate hazard ratios (HRs) and 95% confidence intervals (CIs) for all-cause mortality. Multivariable models were adjusted for baseline clinical, metabolic, and metastatic confounders. Survival curves were generated using the Simon–Makuch method. **Results:** During a median follow-up of 10.0 months (interquartile range: 2.9–30.2 months), 109 patients (14.6%) died. In multivariable time-dependent Cox regression analysis, high-dose IVC therapy (HR: 0.561, 95% CI: 0.319–0.986, *p* = 0.045) and mistletoe therapy (HR: 0.463, 95% CI: 0.231–0.927, *p* = 0.030) were independently associated with significantly reduced overall mortality risk. Hyperthermia therapy showed no significant association with mortality after multivariable adjustment (HR: 1.497, 95% CI: 0.794–2.820, *p* = 0.212). **Conclusions:** High-dose IVC and mistletoe therapies were independently associated with lower overall mortality in female breast cancer patients. These findings support further investigation of these modalities as potential integrative adjuncts to standard care.

## Simple Summary

Complementary therapies are widely used in palliative cancer care, yet prospective evidence evaluating their specific impact on overall survival in female breast cancer patients remains limited. In this prospective cohort study of 748 female breast cancer patients, we evaluated the long-term impact of individual complementary modalities on all-cause mortality. Over the follow-up period, high-dose intravenous vitamin C (IVC) therapy and mistletoe extract therapy were independently associated with a significantly lower risk of overall mortality, even after adjustment for key clinical confounders, including distant metastasis and metabolic factors. Other modalities, including hyperthermia, showed no significant independent association with survival after multivariable adjustment. These findings suggest that high-dose IVC and mistletoe therapies may serve as valuable adjuncts in integrative breast cancer care, warranting validation in future randomized controlled trials.

## 1. Introduction

Breast cancer remains one of the most frequently diagnosed malignancies and a leading cause of cancer-related mortality worldwide, with a particularly rising burden reported in Asian populations [1]. Despite substantial advances in conventional oncologic modalities—including surgery, chemotherapy, radiotherapy, and targeted therapy—long-term disease management continues to pose significant clinical challenges. Even among patients who achieve clinical remission, persistent concerns regarding occult residual disease, systemic recurrence, and treatment-related toxicity continue to compromise survival outcomes and quality of life. Consequently, interest among both patients and healthcare providers in integrating complementary and integrative interventions to address these unmet clinical needs has grown steadily, particularly in advanced disease stages [2,3,4,5].

Complementary therapies in oncology are primarily employed to enhance host immune surveillance, alleviate treatment-induced toxicities, and mitigate chronic systemic inflammation [2,6]. Immunomodulatory agents such as mistletoe extract (*Viscum album* L.) and thymosin alpha-1 aim to restore cell-mediated immunity by enhancing natural killer cell activity and T-lymphocyte proliferation [7,8,9,10]. Concurrently, high-dose intravenous vitamin C (IVC) acts as a potent pro-oxidant at pharmacological concentrations, selectively inducing oxidative stress in tumor cells while exerting anti-inflammatory and mitochondria-protective effects in healthy tissue [11,12,13,14]. Other modalities, such as hyperthermia and hyperbaric oxygen therapy, aim to modify the tumor microenvironment by targeting hypoxia or enhancing thermal chemosensitization [15,16,17,18,19].

Despite their widespread clinical use in palliative and integrative care settings, however, most existing studies have focused predominantly on short-term symptom relief, toxicity reduction, and health-related quality of life (HRQoL) [2,20,21,22,23]. Rigorous longitudinal evidence evaluating whether specific complementary therapies confer tangible overall survival (OS) benefits in female breast cancer patients remains scarce and inconsistent [3,24,25].

To address this critical knowledge gap, we conducted a prospective cohort study to investigate the long-term impact of individual complementary therapeutic modalities on all-cause mortality in female breast cancer patients receiving integrative palliative care. We hypothesized that specific interventions—namely, high-dose IVC and mistletoe therapy—would be independently associated with reduced all-cause mortality, even after rigorous adjustment for key prognostic factors, including tumor metastasis, metabolic comorbidities, and baseline clinicopathological characteristics.

## 2. Materials and Methods

### 2.1. Study Design and Participants

This prospective cohort study analyzed clinical data from female breast cancer patients treated at Yeom Chang Hwan Hospital, a specialized cancer palliative care center in Seoul, South Korea, between April 2011 and May 2024. Eligible participants were identified using the Korean Standard Classification of Diseases code for breast cancer recorded in the hospital’s electronic medical record (EMR) system, with diagnoses subsequently verified through individual chart review. From the initial cohort, patients were excluded if they had incomplete treatment records, fewer than three clinic visits, or a follow-up duration of 45 days or less, as a minimum observation period of 1.5 months was required to establish baseline laboratory values and initiate intervention protocols. Patients lost to follow-up, those with missing administrative records following death, or those affected by EMR system migration were also excluded. After applying these criteria, a final cohort of 748 female breast cancer patients was included in the analysis. Demographic, clinical, and laboratory parameters—including age, body mass index, tumor stage, triple-negative status, recurrence or distant metastasis, family history, and history of conventional oncologic treatment—were extracted from medical records and clinical notes. Metabolic comorbidities, including hypertension, diabetes mellitus, dyslipidemia, hepatic dysfunction, and thyroid disease, were systematically recorded at baseline.

### 2.2. Data Collection and Baseline Measurements

At initial presentation, participants completed a structured questionnaire on lifestyle factors and medical history. Body weight and height were measured in light indoor clothing without shoes, to the nearest 0.1 kg and 0.1 cm, respectively, and body mass index was calculated as weight (kg) divided by height squared (m^2^). Systolic and diastolic blood pressure were measured in the sitting position after 10 min of rest using a standard mercury sphygmomanometer (Baumanometer, W.A. Baum Co., Copiague, NY, USA). Mean arterial pressure was calculated from systolic and diastolic values, and hypertension was defined as systolic blood pressure > 140 mmHg, diastolic blood pressure > 90 mmHg, or current use of antihypertensive medication. After a 12 h overnight fast, venous blood samples were obtained from the antecubital vein. Serum concentrations of total cholesterol, triglycerides, high-density lipoprotein cholesterol, fasting glucose, aspartate aminotransferase, alanine aminotransferase, and gamma–glutamyl transferase were measured enzymatically using automated chemistry analyzers (Hitachi 7600, Hitachi High-Technologies Cor., Tokyo, Japan). Serum C-reactive protein concentration was measured using the ADVIA 1650 chemistry analyzer (Siemens, Tarrytown, NY, USA).

### 2.3. Palliative Care Interventions

Complementary and palliative therapies were administered as adjuvant interventions alongside conventional oncologic treatment, including surgery, chemotherapy, and radiotherapy. High-dose intravenous vitamin C (IVC) was administered once or twice weekly at a dose of 10–100 g (Merit C 10 g, Huons, Jecheon, Republic of Korea); inclusion required a minimum treatment duration of 3 months and more than 10 sessions. Mistletoe extract (Abnova, ABNOBA GmbH, Pforzheim, Germany) was administered subcutaneously at doses of 0.02–20 mg once or twice weekly for at least 3 months and more than 10 sessions [7,8,23]. Thymosin alpha-1 (Alphamosine, containing 1.6 mg thymosin alpha-1; Unione Korea Pharm, Hwasung, Republic of Korea) was administered subcutaneously once or twice weekly for at least 3 months and more than 10 sessions. Cytokine-induced killer (CIK) cell therapy was performed using autologous mononuclear cells isolated from 120 mL of peripheral blood, cultured for 12–21 days with interleukin-2 and immobilized anti-CD3 monoclonal antibodies (Immuncell, GC Cell, Yongin, Republic of Korea); a mean of 6.4 × 10^9^ cells was re-infused over more than 5 sessions. Localized hyperthermia was applied at 40 °C once or twice weekly using the Alba ON 4000 system (Medlogix Srl, Rome, Italy), a device specifically designed for breast cancer treatment, for more than 10 sessions. Additional therapies, including hyperbaric oxygen therapy, alpha-lipoic acid injection, and glutathione injection, were documented based on cumulative treatment history.

### 2.4. Study Outcomes

The primary outcome was overall mortality. Mortality status and exact date of death were verified through clinical records, death certificates, and institutional follow-up logs up to the study endpoint. Follow-up duration was calculated from the date of initial clinic registration to the date of death or last documented clinical contact. No patients experienced severe treatment-related toxicities attributable to high-dose IVC or mistletoe therapy, and no treatment-related deaths occurred [23,26].

### 2.5. Statistical Analysis

Continuous variables are expressed as mean ± standard deviation, and categorical variables as number (percentage). Differences in baseline clinical and laboratory parameters between the survival and death groups were evaluated using one-way analysis of variance for continuous variables and Pearson’s chi-square test for categorical variables. Because exposure to each complementary therapy was not present at baseline but began at a variable time after diagnosis, all analyses of therapy exposure were conducted using a time-dependent covariate framework, with each patient classified as unexposed until the date of first administration of the respective therapy and as exposed thereafter [27]. Survival curves according to complementary therapy exposure were generated using the Simon–Makuch method, an extension of the Kaplan–Meier method that accounts for time-dependent exposure status, and differences between curves were assessed with the Mantel–Byar test, a log-rank test extension for time-dependent covariates [27]. To determine the independent association between each palliative therapy modality and overall mortality, univariate and multivariable Cox proportional-hazards regression models incorporating therapy exposure as a time-dependent covariate were constructed using the counting-process (start-stop) data structure to estimate hazard ratios and 95% confidence intervals [27].

The proportional-hazards assumption for each Cox model was assessed using Schoenfeld residuals, and no significant violation was observed for any covariate (all *p* > 0.05). All statistical analyses were performed using R software (version 4.4.2, R Foundation for Statistical Computing, Vienna, Austria) and/or IBM SPSS Statistics (version 29.0, IBM Corp., Armonk, NY, USA), and a two-tailed *p*-value < 0.05 was considered statistically significant.

## 3. Results

### 3.1. Baseline Characteristics

A total of 748 female patients with breast cancer were included in the final analysis. During a median follow-up of 10.0 months (interquartile range [IQR]: 2.9–30.2 months; mean ± SD: 20.2 ± 24.2 months), 109 patients (14.6%) died, while 639 patients (85.4%) survived. Baseline demographic and clinical characteristics according to survival status are summarized in Table 1 and Table 2. The mean age of the overall population was 55.58 ± 8.57 years, with no significant difference between the survival group (55.70 ± 8.64 years) and the death group (54.89 ± 8.18 years, *p* = 0.346). Similarly, body mass index (BMI) did not differ significantly between groups (22.08 ± 3.19 vs. 22.06 ± 2.97 kg/m^2^, *p* = 0.951).

However, patients in the death group had a significantly higher prevalence of distant metastasis at baseline compared with the survival group (70.6% vs. 17.7%, *p* < 0.001). The proportions of patients with triple-negative breast cancer (36.7% vs. 17.8%, *p* < 0.001), history of chemotherapy (91.7% vs. 67.1%, *p* < 0.001), surgical intervention (69.7% vs. 92.3%, *p* < 0.001), and radiotherapy (64.2% vs. 78.6%, *p* = 0.002) also differed significantly between the two groups. Among laboratory parameters, serum liver enzyme levels were significantly higher in the death group, including aspartate aminotransferase (AST: 43.11 ± 46.18 vs. 25.82 ± 25.59 IU/L, *p* = 0.004) and alanine aminotransferase (ALT: 33.92 ± 30.27 vs. 25.44 ± 22.93 IU/L, *p* = 0.032). Hemoglobin levels were significantly lower in the death group than in the survival group (11.45 ± 1.68 vs. 12.20 ± 1.38 g/dL, *p* < 0.001). Regarding complementary and palliative therapies (Table 2), high-dose intravenous vitamin C (IVC) therapy was received by a significantly higher proportion of patients in the survival group than in the death group (74.3% vs. 62.4%, *p* = 0.014). Conversely, hyperthermia therapy was received by a significantly higher proportion of patients in the death group than in the survival group (55.0% vs. 46.8%, *p* = 0.010). No significant between-group differences were observed for hyperbaric oxygen therapy, mega-vitamin, mistletoe extract, thymosin alpha-1, alpha-lipoic acid, glutathione, or cytokine-induced killer cell therapy in univariate baseline comparisons.

### 3.2. Association Between Complementary Therapies and Overall Mortality

Table 3 presents the results of univariate and multivariable Cox proportional-hazards regression analyses with time-dependent covariates evaluating the association between each complementary modality and overall mortality risk. In the univariate analysis, high-dose IVC therapy (hazard ratio [HR]: 0.557, 95% confidence interval [CI]: 0.356–0.871, *p* = 0.010) and mistletoe therapy (HR: 0.508, 95% CI: 0.271–0.956, *p* = 0.036) were both significantly associated with reduced overall mortality risk. Conversely, hyperthermia therapy (HR: 1.141, 95% CI: 0.724–1.796, *p* = 0.570) showed no statistically significant association in the univariate model.

After multivariable adjustment using time-dependent covariates—including age, metastasis, triple-negative status, chemotherapy, operation, radiotherapy, and other palliative modalities—both high-dose IVC therapy and mistletoe therapy remained independent protective factors against overall mortality. Specifically, high-dose IVC therapy was associated with a 43.9% reduction in mortality risk (multivariable HR: 0.561, 95% CI: 0.319–0.986, *p* = 0.045). Similarly, mistletoe therapy was associated with a 53.7% reduction in mortality risk (multivariable HR: 0.463, 95% CI: 0.231–0.927, *p* = 0.030). Hyperthermia therapy showed no statistically significant association with overall mortality after multivariable adjustment (HR: 1.497, 95% CI: 0.794–2.820, *p* = 0.212). Other complementary interventions—including hyperbaric oxygen therapy, thymosin alpha-1, alpha-lipoic acid, glutathione, and Cytokine-induced killer cell therapy—also showed no statistically significant association with overall mortality in the multivariable model. Simon–Makuch survival curves taking into account time-dependent exposure demonstrated significantly improved overall survival for patients receiving high-dose IVC therapy compared with unexposed patients (Mantel–Byar test, *p* < 0.001; Figure 1), as well as for patients receiving mistletoe therapy compared with unexposed patients (Mantel-Byar test, *p* = 0.001; Figure 2).

## 4. Discussion

This prospective cohort study evaluated the long-term impact of various complementary and palliative therapies on overall survival among 748 female patients with breast cancer. Our primary findings show that high-dose intravenous vitamin C (IVC) therapy and mistletoe extract therapy were independently associated with a significant reduction in overall mortality risk, even after comprehensive adjustment for clinical confounders, including tumor metastasis, baseline metabolic factors, and organ function markers. Specifically, high-dose IVC therapy was associated with a 43.9% reduction in overall mortality risk (HR: 0.561, 95% CI: 0.319–0.986, *p* = 0.045), while mistletoe therapy was associated with a 53.7% risk reduction (HR: 0.463, 95% CI: 0.231–0.927, *p* = 0.030). Conversely, although hyperthermia therapy showed an increased hazard ratio in univariate analysis, this association lost statistical significance in the multivariable model (HR: 1.497, 95% CI: 0.794–2.820, *p* = 0.212), suggesting substantial confounding by baseline disease severity.

The survival association observed with high-dose IVC therapy may be explained by several pharmacological and cellular mechanisms. Pharmacokinetic studies indicate that intravenous administration achieves millimolar plasma concentrations exceeding 20 mmol/L, levels unachievable through oral supplementation [11,13]. At these pharmacological concentrations, ascorbate acts as a pro-oxidant by mediating metal-catalyzed generation of extracellular hydrogen peroxide (H_2_O_2_) [12,13,14]. Malignant cells may display preferential susceptibility to IVC-induced oxidative cytotoxicity due to lower catalase activity, deficient glutathione peroxidase expression, and impaired redox homeostasis [12,13,14]. IVC has also been reported to enhance mitochondrial oxidative phosphorylation, modulate pro-inflammatory cytokine expression, and serve as a cofactor for Ten-Eleven Translocation (TET) dioxygenases, thereby supporting epigenetic regulation through DNA demethylation [11,14,28]. Clinically, our findings are consistent with reports by Günes-Bayir and Kiziltan and by Yeom et al., who described functional improvements and survival extension in patients receiving high-dose IVC [21,29], as well as with recent evidence supporting integrative high-dose ascorbate protocols [24].

Mistletoe therapy (*Viscum album* L.) is thought to exert multi-targeted anti-neoplastic effects primarily through immunomodulatory and cytotoxic pathways. Active constituents, including mistletoe lectins and viscotoxins, have been reported to stimulate cell-mediated immunity by enhancing natural killer cell activity, promoting T-lymphocyte proliferation, and increasing pro-inflammatory cytokine release [7,8]. Beyond immune activation, mistletoe extracts may induce caspase-dependent intrinsic and extrinsic apoptotic pathways while downregulating vascular endothelial growth factor (VEGF), thereby inhibiting tumor angiogenesis [7,8,10]. These mechanistic findings are consistent with our results and with long-term observational studies by Grossarth-Maticek and Ziegler [30] and Tröger et al. [31], which reported survival advantages in cancer patients receiving mistletoe extract, as well as with recent randomized trials evaluating mistletoe therapy in breast cancer [25].

The positive association observed between hyperthermia therapy and mortality in the univariate analysis warrants careful interpretation. Hyperthermia is frequently prescribed as a salvage intervention for patients with advanced, recurrent, or treatment-refractory disease [15,17]. This indication bias is reflected in our baseline data, in which hyperthermia recipients had higher mortality rates prior to multivariable adjustment. After adjusting for metastatic burden and systemic inflammatory status, hyperthermia was no longer an independent predictor of mortality. Biologically, reduced thermal efficacy in advanced malignancy may also involve thermotolerance driven by heat-shock protein induction (e.g., HSP70, HSP90) and altered perfusion within hypoxic tumor microenvironments [16,18].

A major strength of this study is its prospective, longitudinal design in a large, well-characterized cohort, allowing for comparative survival analysis across multiple complementary therapies simultaneously [3,4,5]. Crucially, we rigorously eliminated potential immortal time bias in this updated analysis by reformulating all complementary therapy exposures into a time-dependent covariate framework using a counting-process model [27]. Under this framework, person-time prior to meeting treatment exposure thresholds was appropriately assigned to the unexposed state, preserving analytical integrity [27]. Several limitations should nonetheless be acknowledged.

First, because this was a non-randomized, real-world cohort in which treatment modality was determined by patient preference and clinical judgment, residual confounding by indication cannot be excluded; patients who chose or tolerated IVC or mistletoe therapy may have differed systematically from non-recipients in baseline performance status, health-seeking behavior, or underlying disease trajectory.

Second, although our initial exposure protocol required a minimum treatment duration, we rigorously addressed potential immortal time bias in this revised analysis by reformulating all complementary therapy exposures into a time-dependent covariate framework using a counting-process model. Under this approach, protective associations remained statistically significant for high-dose IVC and mistletoe therapy. However, as an observational study, prospective randomized controlled trials remain necessary to establish definitive causality. Third, detailed continuous cumulative dose–response relationships and exact long-term administration schedules were not modeled, due to the inherent constraints of real-world EMR documentation. Although we applied a strict minimum exposure threshold to exclude sporadic or inadequate use, future prospective trials with standardized dosing protocols are warranted to establish the optimal dose–survival response relationship. Fourth, detailed records regarding specific lines of systemic targeted agents—such as endocrine therapy, HER2-targeted drugs, and CDK4/6 inhibitors—were not comprehensively captured in our EMR database. Although we adjusted for triple-negative breast cancer (TNBC) status, chemotherapy, surgical resection, and radiotherapy as key clinical proxies for systemic treatment intensity, residual confounding from unmeasured specific regimens cannot be completely ruled out. Fifth, detailed T and N staging contained substantial missing data (>40%) in EMR records and were omitted from multivariable adjustment to avoid listwise deletion bias; instead, distant metastasis status—which was 100% verified—was utilized as the primary systemic stage indicator. Finally, as a single-center study conducted in a specialized integrative palliative care setting in Korea, generalizability to other populations and healthcare settings may be limited.

In summary, this prospective cohort study found that high-dose IVC and mistletoe therapies were independently associated with lower overall mortality in female breast cancer patients. These findings support further investigation of complementary modalities as potential adjuncts to standard oncologic care [2,3,5]. To overcome the inherent constraints of observational EMR datasets, establishing high-precision prospective real-world data registries and conducting randomized controlled trials will be critical to confirm causality, establish standardized treatment protocols, and guide clinical decision-making [32].

## 5. Conclusions

In conclusion, high-dose intravenous vitamin C and mistletoe extract therapies were independently associated with significantly reduced overall mortality in female breast cancer patients receiving palliative care. These associations remained robust after comprehensive multivariable adjustment for clinical, metabolic, and metastatic confounders. Our findings suggest that these complementary interventions may serve as promising adjuncts to conventional cancer care, warranting further investigation as potential contributors to long-term survival. Prospective, ideally randomized, clinical studies are needed to confirm these findings, clarify causality, and establish standardized treatment protocols.

## Figures and Tables

**Figure 1:**
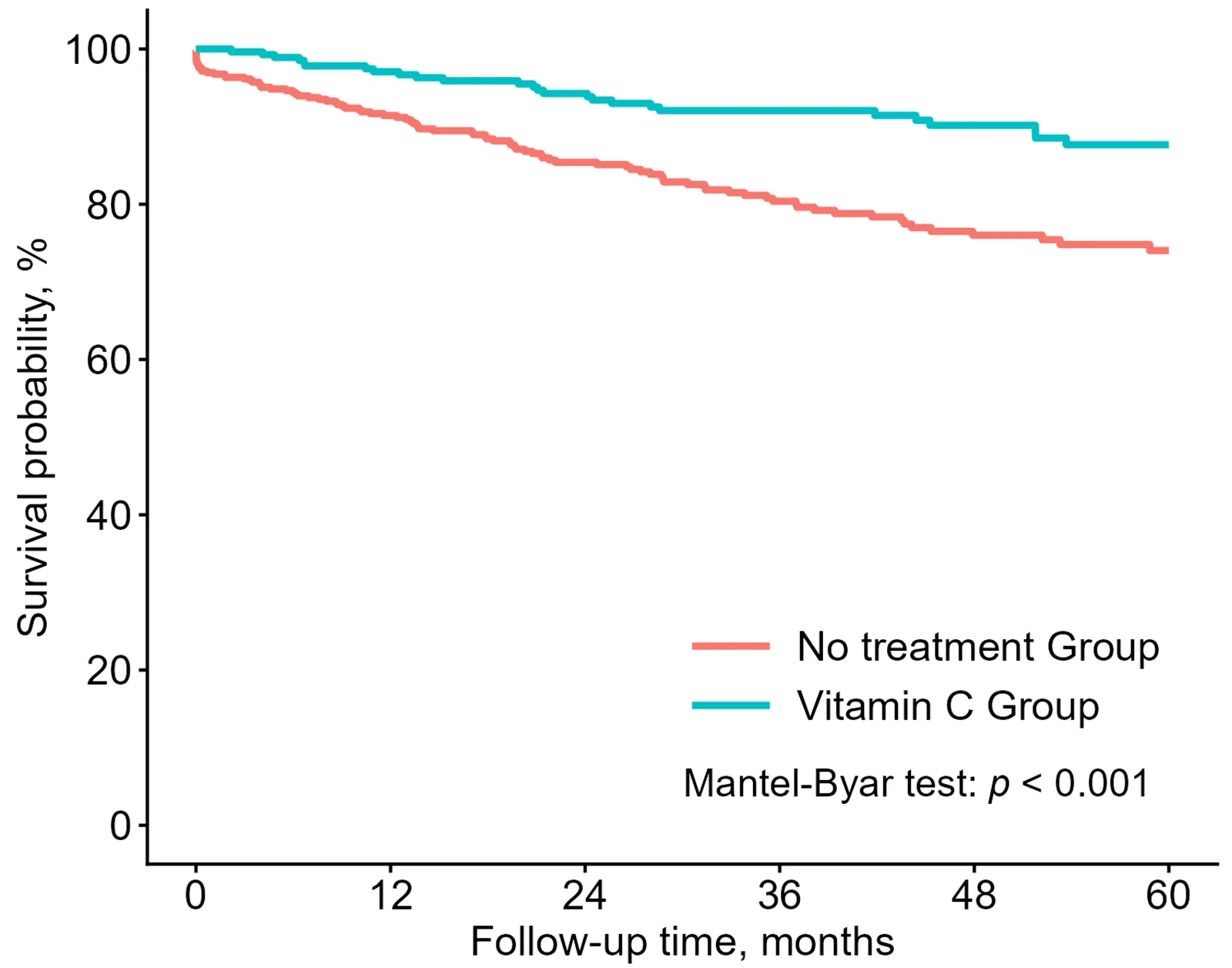
Simon–Makuch survival curves comparing breast cancer patients according to time-dependent exposure to high-dose intravenous vitamin C (IVC) therapy (blue line, exposed) versus no IVC therapy (red line, unexposed). Patients exposed to high-dose IVC showed a trend toward improved long-term overall survival over a 60-month follow-up period (Mantel–Byar test, *p* < 0.001).

**Figure 2:**
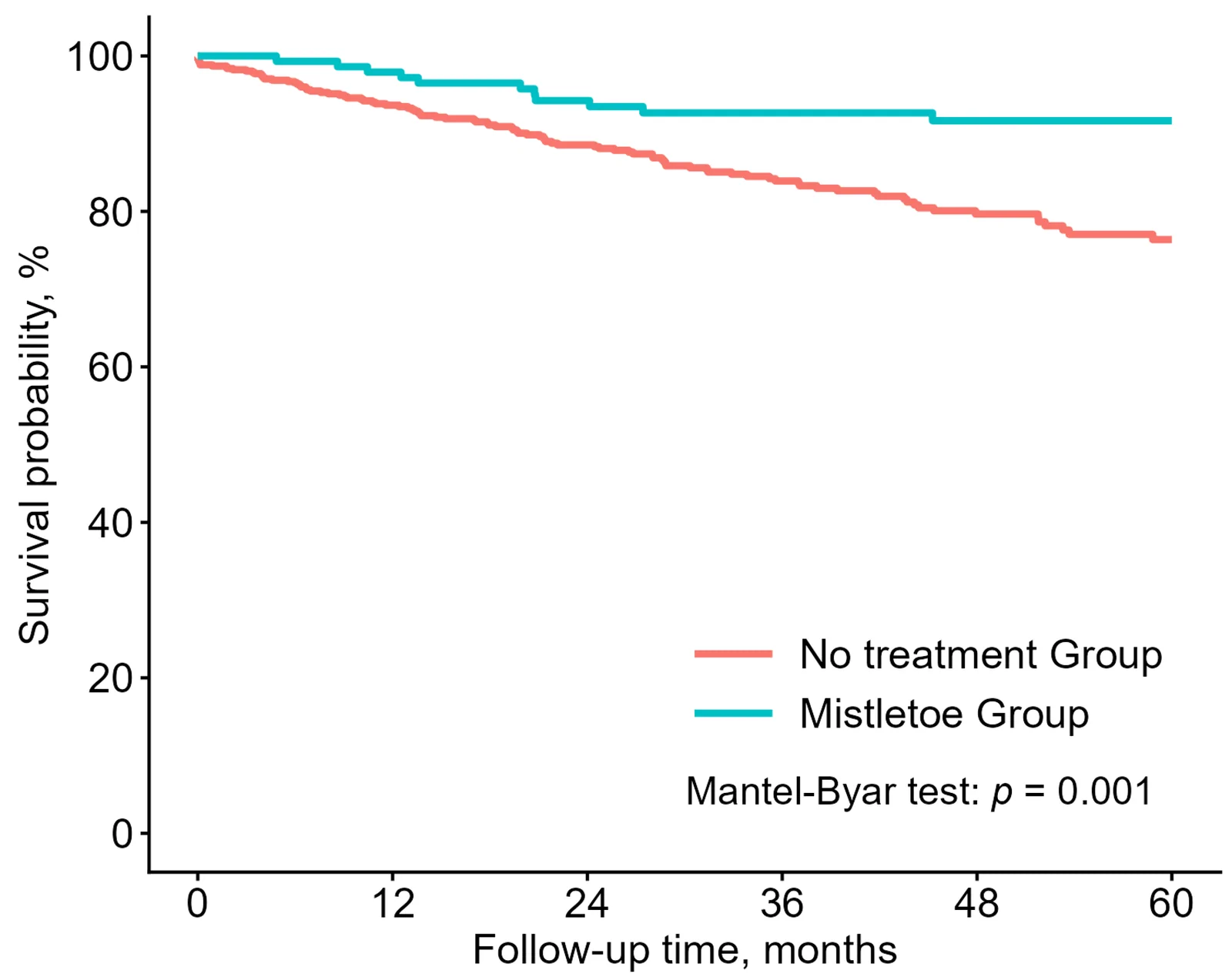
Simon–Makuch survival curves comparing breast cancer patients according to time-dependent exposure to mistletoe extract therapy (blue line, exposed) versus no mistletoe therapy (red line, unexposed). Exposure to mistletoe therapy was associated with significantly superior overall survival compared to no exposure over the 60-month follow-up period (Mantel–Byar test, *p* = 0.001).

**Table 1:** Baseline characteristics of the study population according to the mortality in breast cancer patients.

Patients with Breast Cancer	Overall (*n* = 748)	Survive (*n* = 639)	Death (*n* = 109)	*p*-Value ^a^
Age	55.58 ± 8.57	55.70 ± 8.64	54.89 ± 8.18	0.346
Body mass index (kg/m^2^)	22.08 ± 3.16	22.08 ± 3.19	22.06 ± 2.97	0.951
Metastasis *n* (%)	190 (25.4%)	113 (17.7%)	77 (70.6%)	<0.001
Triple Negative	154 (20.6%)	114 (17.8%)	40 (36.7%)	<0.001
Chemotherapy	529 (70.7%)	429 (67.1%)	100 (91.7%)	<0.001
Operation	666 (89.0%)	590 (92.3%)	76 (69.7%)	<0.001
Radiotherapy	572 (76.5%)	502 (78.6%)	70 (64.2%)	0.002
Total cholesterol (mg/dL)	189.33 ± 41.76	187.77 ± 39.55	199.97 ± 53.63	0.094
Triglyceride (mg/dL)	126.27 ± 89.80	126.57 ± 93.77	124.35 ± 59.38	0.812
HDL-cholesterol (mg/dL)	56.18 ± 15.51	56.63 ± 15.14	53.40 ± 17.59	0.195
Aspartate aminotransferase (IU/L)	28.16 ± 29.77	25.82 ± 25.59	43.11 ± 46.18	0.004
Alanine aminotransferase (IU/L)	26.59 ± 24.19	25.44 ± 22.93	33.92 ± 30.27	0.032
BUN	13.71 ± 4.50	13.72 ± 4.39	13.65 ± 5.18	0.924
Creatinine (mg/dL)	0.69 ± 0.76	0.69 ± 0.82	0.64 ± 0.14	0.212
White blood cell count (/μL)	5283.28 ± 4535.76	5338.00 ± 4743.91	4925.91 ± 2821.72	0.323
Hemoglobin (g/dL)	12.10 ± 1.45	12.20 ± 1.38	11.45 ± 1.68	<0.001
C-reactive protein (mg/L)	1.55 ± 10.31	1.59 ± 11.08	1.26 ± 2.56	0.630
Hypertension (%)	37 (4.9%)	36 (5.6%)	1 (0.9%)	0.063
Diabetes mellitus (%)	23 (3.1%)	23 (3.6%)	0 (0.0%)	0.087

^a^ Differences in baseline clinical and laboratory parameters between the survival and death groups were evaluated using one-way analysis of variance for continuous variables and Pearson’s chi-square test for categorical variables.

**Table 2:** Palliative care of the study population according to the mortality in breast cancer patients.

Patients with Breast Cancer	Overall (*n* = 748)	Survive (*n* = 639)	Death (*n* = 109)	*p*-Value ^a^
Hyperbaric oxygen therapy *n* (%)	372 (49.7%)	319 (49.9%)	53 (48.6%)	0.883
Mega-vitamin *n* (%)	534 (71.4%)	462 (72.3%)	72 (66.1%)	0.223
High Dose Vitamin C *n* (%)	543 (72.6%)	475 (74.3%)	68 (62.4%)	0.014
Mistletoe *n* (%)	224 (29.9%)	197 (30.8%)	27 (24.8%)	0.245
Thymosin alpha-1	447 (59.8%)	376 (58.8%)	71 (65.1%)	0.257
Lipoic Acid Injection	195 (26.1%)	172 (26.9%)	23 (21.1%)	0.246
Glutathione Injection	375 (50.1%)	321 (50.2%)	54 (49.5%)	0.976
Cytokine-induced killer cell therapy	118 (15.8%)	97 (15.2%)	21 (19.3%)	0.347
Hyperthermia Therapy	359 (48.0%)	299 (46.8%)	60 (55.0%)	0.010

^a^ Differences in baseline clinical and laboratory parameters between the survival and death groups were evaluated using one-way analysis of variance for continuous variables and Pearson’s chi-square test for categorical variables.

**Table 3:** Multivariable Cox proportional-hazards regression models with time-dependent covariates for overall mortality according to palliative care exposure.

Variables	Univariate		Multivariate	
	HR (95% CI)	*p*-Value	HR (95% CI)	*p*-Value
Age	0.985 (0.963–1.008)	0.208	0.991 (0.969–1.014)	0.464
Metastasis	7.733 (5.060–11.816)	<0.001	6.026 (3.769–9.632)	<0.001
Triple Negative	3.114 (2.084–4.652)	<0.001	3.342 (2.161–5.169)	<0.001
Chemotherapy	4.567 (2.302–9.059)	<0.001	1.909 (0.927–3.931)	0.079
Operation	0.286 (0.186–0.439)	<0.001	0.491 (0.284–0.849)	0.011
Radiotherapy	0.622 (0.413–0.936)	0.023	1.051 (0.643–1.720)	0.842
Hyperbaric oxygen therapy	1.011 (0.643–1.591)	0.961	1.039 (0.560–1.927)	0.904
High Dose Vitamin C	0.557 (0.356–0.871)	0.010	0.561 (0.319–0.986)	0.045
Mistletoe	0.508 (0.271–0.956)	0.036	0.463 (0.231–0.927)	0.030
Thymosin alpha-1	0.786 (0.505–1.225)	0.288	0.957 (0.538–1.703)	0.881
Lipoic Acid Injection	0.742 (0.386–1.428)	0.372	0.879 (0.395–1.956)	0.752
Glutathione Injection	1.155 (0.742–1.796)	0.524	1.392 (0.797–2.430)	0.245
Cytokine-induced killer cell therapy	1.117 (0.562–2.220)	0.752	1.114 (0.526–2.357)	0.778
Hyperthermia Therapy	1.141 (0.724–1.796)	0.570	1.497 (0.794–2.820)	0.212

## Data Availability

The data presented in this study are available on request from the corresponding author.

## Abbreviations

The following abbreviations are used in this manuscript:

ALT	Alanine aminotransferase
ANOVA	Analysis of variance
AST	Aspartate aminotransferase
BMI	Body mass index
BUN	Blood urea nitrogen
CI	Confidence interval
CIK	Cytokine-induced killer
CRP	C-reactive protein
EMR	Electronic medical record
HDL	High-density lipoprotein
HR	Hazard ratio
HRQoL	Health-related quality of life
HSP	Heat-shock protein
IVC	Intravenous vitamin C
OS	Overall survival
TET	Ten-Eleven Translocation
VEGF	Vascular endothelial growth factor
